# Krill Hotspot Formation and Phenology in the California Current Ecosystem

**DOI:** 10.1029/2020GL088039

**Published:** 2020-06-28

**Authors:** Jerome Fiechter, Jarrod A. Santora, Francisco Chavez, Devon Northcott, Monique Messié

**Affiliations:** ^1^ Ocean Sciences Department University of California Santa Cruz CA USA; ^2^ Fisheries Ecology Division, Southwest Fisheries Science Center, National Marine Fisheries Service National Oceanic and Atmospheric Administration Santa Cruz CA USA; ^3^ Department of Applied Mathematics University of California Santa Cruz CA USA; ^4^ Monterey Bay Aquarium Research Institute Moss Landing CA USA; ^5^ Now at Scripps Institution of Oceanography University of California, San Diego La Jolla CA USA

**Keywords:** California Current, ecosystem hotspots, coastal upwelling, krill, biophysical model, top predators

## Abstract

In the California Current Ecosystem, krill represent a key link between primary production and higher trophic level species owing to their central position in the food web and tendency to form dense aggregations. However, the strongly advective circulation associated with coastal upwelling may decouple the timing, occurrence, and persistence of krill hotspots from phytoplankton biomass and nutrient sources. Results from a coupled physical‐biological model provide insights into fundamental mechanisms controlling the phenology of krill hotspots in the California Current Ecosystem, and their sensitivity to alongshore changes in coastal upwelling intensity. The simulation indicates that dynamics controlling krill hotspot formation, intensity, and persistence on seasonal and interannual timescales are strongly heterogeneous and related to alongshore variations in upwelling‐favorable winds, primary production, and ocean currents. Furthermore, regions promoting persistent krill hotspot formation coincide with increased observed abundance of top predators, indicating that the model resolves important ecosystem complexity and function.

## Key Points

- Krill hotspot formation and persistence are strongly heterogeneous in space and time
- Krill hotspots are shaped by local upwelling variability and regional circulation
- Krill hotspots coincide with observed distributions of marine mammals and seabirds

## 1 Introduction

In marine ecosystems, linkages between planktonic organisms, forage fish, and predators must be considered in a context where ocean currents continuously reshape and rescale the underlying physical and biological environments. While, in theory, bottom‐up processes influencing local nutrient enrichment and retention lead to sustained aggregations of mid and high trophic level species (Bakun, 1996; Santora et al., 2017), the formation and persistence of ecosystem hotspots in regions dominated by a strongly advective ocean circulation may become decoupled in space and time from nutrient sources and primary production (Messié & Chavez, 2017), making it difficult to understand trophic transfer of energy and predict aggregation locations.

In the California Current Ecosystem (CCE), one of the four major eastern boundary current upwelling regions, zooplankton species represent a key link between primary production and higher trophic level species. In particular, krill (euphausiids) hotspots, defined here as reoccurring areas of patchy high concentrations that range in size from tens to hundreds of kilometers and persist over weeks to months, often coincide with increased abundance of higher trophic level organisms, including commercially harvested species (Santora et al., 2011; Santora, Dorman, et al., 2017; Santora, Sydeman, et al., 2017). Furthermore, growth and reproduction of krill in the CCE are linked to seasonal variability of upwelling (i.e., the primary mechanism supplying nutrients that support primary production) and advective properties of the ocean circulation (Brinton, 1962b; Brinton & Townsend, 2003; Croll et al., 2005; Messié & Chavez, 2017). Subsequent aggregation and retention of these organisms are influenced by local physical processes and topographic features (Bakun, 1996; Santora et al., 2018; Woodson & Litvin, 2014), thereby mediating the potential formation of ecosystem hotspots by controlling their distribution and availability to higher trophic level species. During the upwelling season, the dominant circulation features controlling advective properties in the CCE are the strong alongshore (equatorward) coastal jet associated with nearshore upwelling, mesoscale processes in the transition zone, and the slower meandering flow of the California Current proper offshore (Checkley & Barth, 2009).

While satellites have greatly expanded the ability to concurrently observe atmospheric conditions, surface physics, and primary production over a wide range of spatial and temporal scales (Edwards et al., 2010), they provide limited information on the occurrence, structure, and underlying biophysical processes associated with ecosystem hotspots involving midwater forage species. This is especially true of pelagic ecosystems where foundational species, such as zooplankton and schooling forage fish, form dense aggregations and have population dynamics intensely subject to climate variability (Chavez et al., 2003; Lindegren et al., 2013; Rykaczewski & Checkley, 2008). Coupled biophysical models, combined with observational studies, offer a promising way forward to elucidate mechanisms associated with ecosystem hotspot formation, phenology, and sensitivity to climate change (Cury et al., 2008). In the CCE, recent modeling approaches, parameterized and evaluated with existing observations, have been used to assess environmental drivers of variability in various planktonic organisms (Dorman, Sydeman, Garcia‐Reyes, Zeno, & Santora, 2015; Fiechter et al., 2018; Messié & Chavez, 2017; Santora, Dorman, & Sydeman, 2017).

This study expands on previous modeling work by revealing how scales of physical advection combine with biological responses to sustain seasonal formation and persistence of krill hotspots, and their connections to alongshore variation in coastal upwelling intensity. Importantly, the model solution is compared with fisheries ecosystem surveys that monitor krill fluctuations and distribution patterns of top predators, leading to broader considerations on the ecosystem implications of resolving seasonal krill hotspots in the CCE and other krill centric systems.

## 2 Methods

The numerical model is an implementation of the Regional Ocean Modelling System (ROMS; Haidvogel et al., 2008; Shchepetkin & McWilliams, 2005) coupled to NEMUCSC, a customized version of the North Pacific Ecosystem Model for Understanding Regional Oceanography (NEMURO; Fiechter et al., 2018; Kishi et al., 2007). NEMUCSC includes three limiting macronutrients (nitrate, ammonium, and silicic acid), two phytoplankton (nanophytoplankton and diatoms) and three zooplankton (micro, meso, and predatory) functional groups, and three detritus pools (dissolved and particulate organic nitrogen and particulate silica). The predatory zooplankton group is parameterized to represent *Euphausia pacifica*, the numerically dominant euphausiid in the CCE (Brinton, 1962a, 1962b; Lavaniegos & Ohman, 2007). While NEMURO originally used “predatory zooplankton” as a broad group including gelatinous plankton and euphausiids with uniform grazing rates (0.2 day^−1^ at 0°C) on diatom, microzooplankton, and mesozooplankton, NEMUCSC introduces traits specific to *Euphausia pacifica* by favoring predation on diatoms (0.3 day^−1^ at 0°C) and copepods (0.1 day^−1^ at 0°C; Dorman, Sydeman, Bograd, & Powell, 2015; Ohman, 1984; Table S1 in the supporting information). The reduced direct grazing mortality by predatory zooplankton on microzooplankton and mesozooplankton is accounted for by a fractional increase of their density‐dependent natural mortality.

To improve the representation of regional circulation patterns, the ROMS model downscales a 1/10° (~10 km) data‐assimilative physical reanalysis for the broader CCE to a 1/30° (~3 km) domain for the central CCE (Figure S1 in the supporting information; Fiechter et al., 2018). The 1/30° model is run for 1990–2010, and the analysis focuses on nearshore (0–100 km offshore) variability during peak upwelling season in the central CCE (May–August). Simulated krill concentrations are evaluated with in situ estimates of krill abundance from the NOAA Rockfish Recruitment and Ecosystem Assessment Survey (RREAS; Figure S1): relative abundance is measured as catch‐per‐unit‐effort from midwater trawls during May–June 1990–2010, and krill aggregations are determined from concurrent acoustic surveys (starting in 2000), indexed as the nautical area scattering coefficient (m^2^/nmi^2^; Santora et al., 2011, 2018). Additional evaluation of physical and biogeochemical fields from the 1/10° and 1/30° model solutions is available in Schroeder et al. (2014), Neveu et al. (2016), and Fiechter et al. (2018).

Hotspots in the model are identified as local alongshore peaks in krill abundance based on zonally averaged (0–100 km offshore) values, limited yearly to periods when monthly mean concentrations exceed the annual mean plus one standard deviation (Santora et al., 2018; Dorman, Sydeman, Garcia‐Reyes, et al., 2015). To provide greater ecological context, the following hotspot properties are subsequently defined annually: “intensity” (maximum krill concentrations), “duration” (number of months during which krill concentrations exceed one standard deviation above the mean), and “peak timing” (month of highest krill concentrations). These properties are calculated over 0.5° alongshore bands centered near the peaks identified using the method described above.

Time series of annual hotspot property (intensity, duration, or peak timing) averaged over each 0.5° alongshore bands are combined into an empirical orthogonal function (EOF) analysis (e.g., Thomson & Emery, 2014) to detect coherent modes of variability across hotspots (EOF spatial modes). The standardized temporal amplitudes of the EOF modes are subsequently used to identify environmental conditions associated with latitudinal hotspot response by contrasting years of positive amplitude (>0.5 standard deviation) and negative amplitude (< −0.5 standard deviation; Figure S2). The environmental variables considered are annual mean alongshore wind stress, vertical velocity at 40 m, depth of 26.0 isopycnal, and nitrate concentration at 60 m during May–August. For example, anomalous vertical velocities for a given EOF mode are calculated by subtracting vertical velocities averaged over negative amplitude years (< −0.5 SD) from those averaged over positive amplitude years (>0.5 SD).

## 3 Results

### 3.1 Patterns and Variability of Krill Aggregations

**Figure 1:**
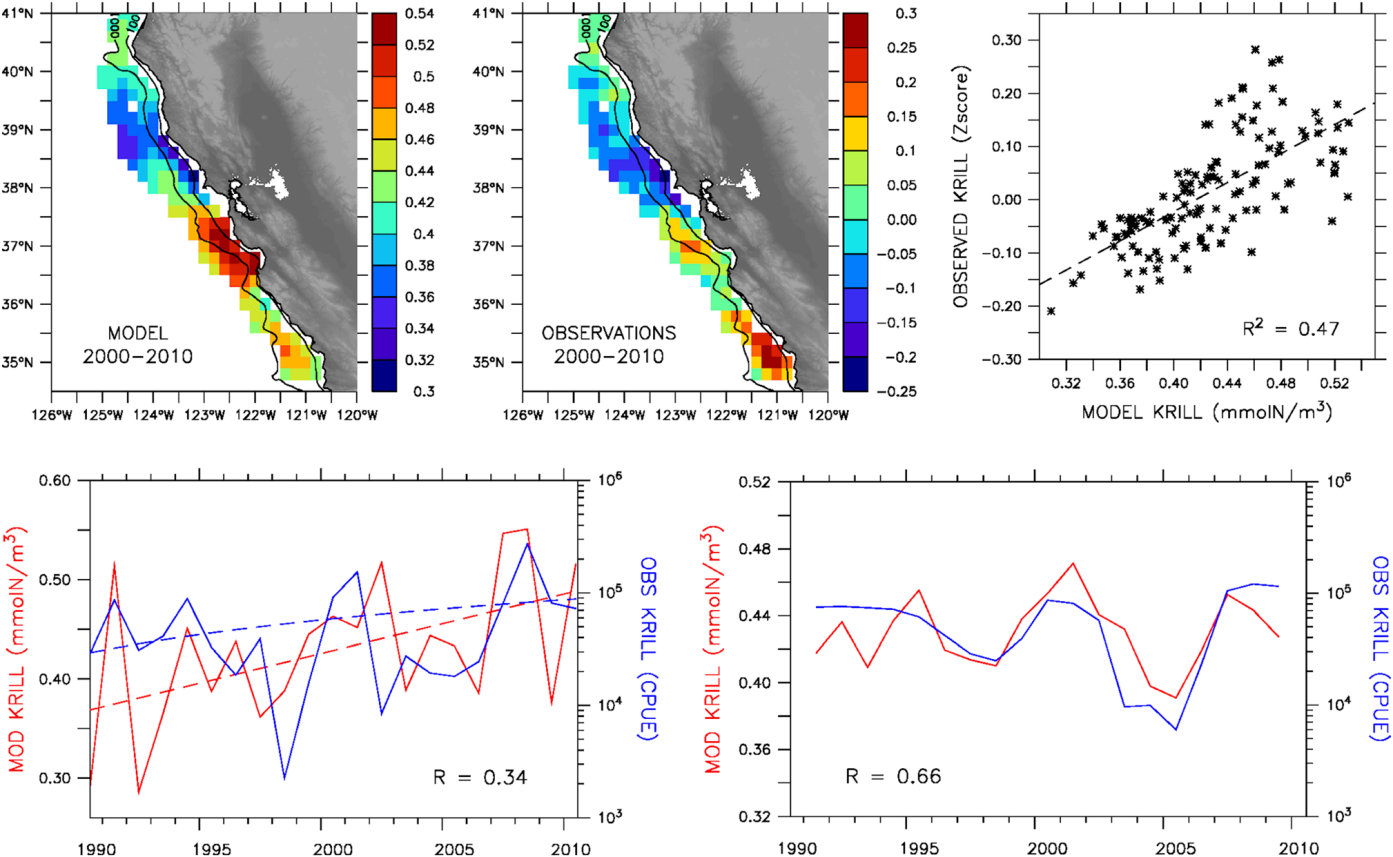
Spatial and temporal krill variability during May–June. Top: Simulated (left; mmolN/m^3^) and observed (center; nautical area scattering coefficient *Z*‐score) mean krill abundances during 2000–2010 at observation locations, and linear regression between simulated and observed values (right); contour lines indicate 100‐m and 1,000‐m isobaths. Bottom: Annual (left) and 3‐year running mean (right) simulated (red; mmolN/m^3^) and observed (blue; catch‐per‐unit‐effort) krill abundances averaged over 36–38°N and 0–100 km offshore during 1990–2010 (running mean is applied to smooth out annual differences while retaining lower frequency variability); the dashed lines in left panel indicate long‐term trends.

During May–June, when in situ krill observations are most consistently available, simulated krill concentrations resolve three distinct accumulation regions: two stronger ones near 35 and 37°N and a weaker one near 40°N. These regions are largely consistent with krill aggregations identified from acoustic surveys (Santora et al., 2011, 2018), although the model underestimates relative intensity at 35°N and overestimates it at 37°N (Figure 1, upper panels). Nonetheless, simulated krill concentrations explain approximately half of the observed spatial variance, lending confidence that the simulation adequately represents alongshore regions favoring persistent krill aggregations. Importantly, variable survey effort within a region and year may impact the assessment of observed krill aggregations estimated from acoustic sampling.

Low‐frequency (3‐year running mean) variability of simulated krill concentrations in the region 36–38°N (approximately Monterey Bay to Gulf of the Farallones) is comparable to observed relative abundance from midwater trawls and exhibits a periodicity of ~6 years and a long‐term increasing trend (Figure 1, lower panels). However, the magnitude of the annual trend is underestimated by the model with an increase of 1.4% of the long‐term mean compared to 5.0% for the observations. Interannual variations are also weaker in the model, owing to the stabilizing effect of the quadratic natural mortality term in NEMUCSC, which prevents exponential fluctuations in phytoplankton and zooplankton biomasses (viz. linear vs. logarithmic *y* axes in Figure 1, lower panels). Year‐to‐year differences are also attributable to inconsistencies between the simulation and in situ sampling; such as those arising from krill patchiness and relative contributions of other krill species (e.g., *Thysanoessa spinifera*) to total abundance. Since the midwater trawl catch‐per‐unit‐effort only provides a measure of relative abundance, simulated concentrations are further evaluated with springtime krill abundance reported by Lavaniegos and Ohman (2007) for central California (Figure S1). Depth‐integrated (surface to 200‐m depth) krill concentrations in the model average to 877 mgC/m^2^ during 1990–2010, compared to a mean observed euphausiid abundance of 294 mgC/m^2^ during 1951–2005 (NEMUCSC nitrogen units are converted to carbon using a C:N of 4 (Lindley et al., 1999)). While the model overestimates the observed mean by a factor of 3, the discrepancy may be partly explained by the increasing trend in krill abundance during 1990–2010, combined with the fact that observations are biased toward 1951–1990 with only six annual springtime values available for 1990–2005.

**Figure 2:**
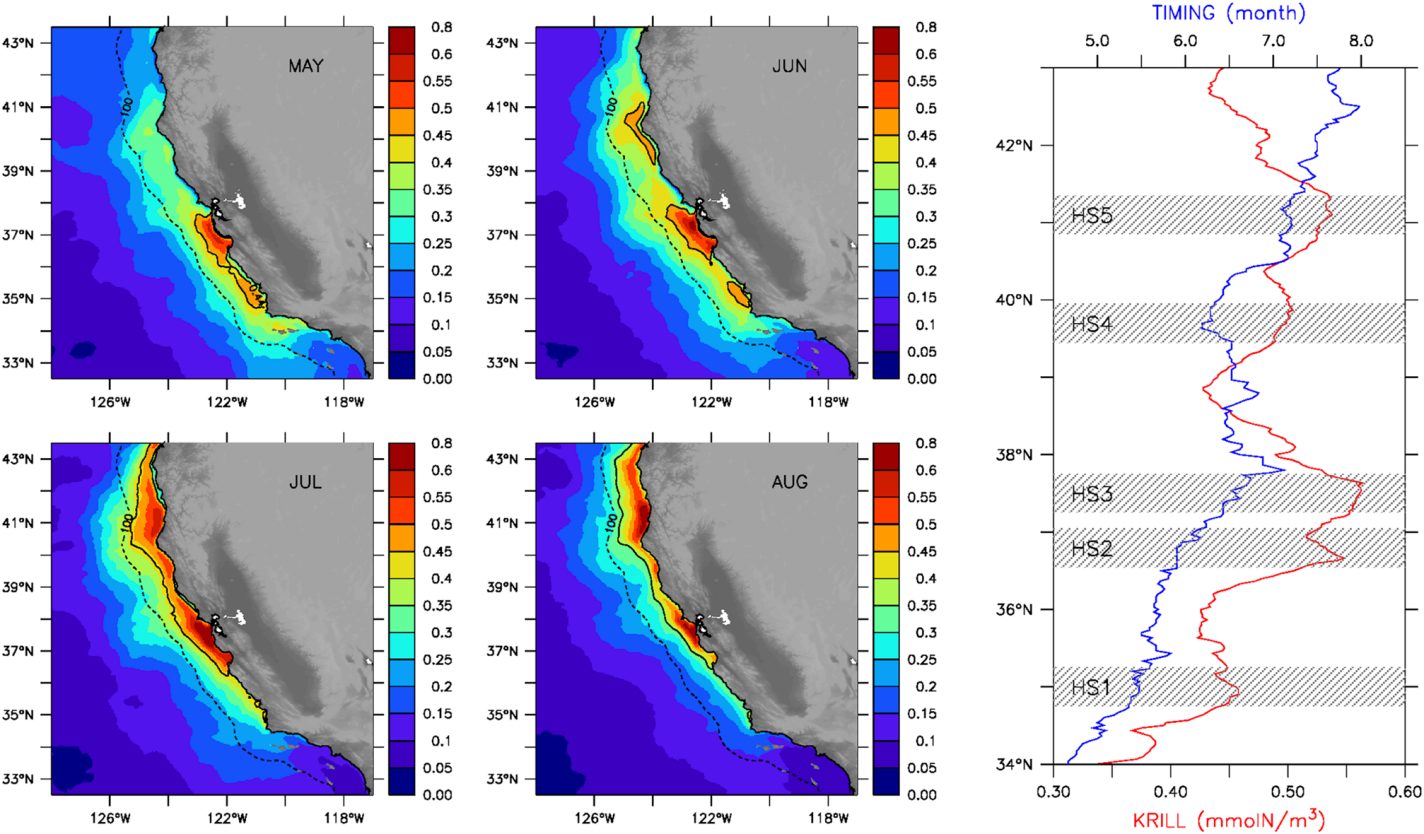
Phenology of krill hotspots. Left and center: Climatological mean simulated surface krill concentrations (mmolN/m^3^) during May, June, July, and August (the dashed contour line indicates 100 km offshore). Right: Simulated mean nearshore surface krill hotspot concentrations (red; mmolN/m^3^) and peak timing (blue; month) as a function of latitude; the shaded 0.5° bands denote alongshore extent of seasonal peaks identified as local hotspots in simulated surface krill concentrations averaged 0–100 km offshore.

The limited spatiotemporal coverage of shipboard surveys off California restricts the ability to determine transient properties of enhanced krill concentration regions. Hence, the simulation is used to explore potential mechanisms controlling the phenology of krill aggregations in the central CCE in response to coastal upwelling variability. The most striking feature in the model solution is the alongshore variation in location and intensity of peak concentrations from May to August under the combined effects of alongshore advection by coastal currents and the seasonal northward progression of upwelling (Fiechter et al., 2018; Messié & Chavez, 2015; Figure 2). In the central and northern parts of the domain, elevated krill concentrations during spring are found near 37 and 40°N, which is approximately 100 km equatorward of where maximum phytoplankton biomasses occur (near 38 and 41°N, respectively), whereas elevated krill concentrations during summer are essentially collocated with regions of peak phytoplankton biomass (Figure S3).

The latitudinal offset between regions of simulated peak phytoplankton and krill biomasses corresponds to the distance local krill concentrations would typically be advected alongshore by near surface currents over a krill doubling period (~1 week in the model), which can reach 50–100 km during spring and decrease to less than 50 km during summer. This finding is dynamically consistent with the seasonal development of the alongshore coastal jet, which forms nearshore during spring and migrates offshore as upwelling strengthens (Strub & James, 2000), resulting in stronger alongshore advection near the coast in May–June relative to July–August. In contrast, peak phytoplankton and krill concentrations near 35°N remain collocated throughout the upwelling months, as alongshore transport in that region is typically weak with a poleward tendency (i.e., advection distances less than ~30 km on average; Figure S3). These latitudinal and seasonal changes in the spatial coupling between krill and their nutrient and prey sources further confirm the importance of surface advection as a key process shaping alongshore distribution of phytoplankton biomass (Fiechter et al., 2018) and krill aggregations (Dorman, Sydeman, Garcia‐Reyes, et al., 2015; Messié & Chavez, 2017) in the CCE.

### 3.2 Intensity, Timing, and Duration of Krill Hotspots

Over the course of the upwelling season, the simulation reveals the occurrence of five distinct hotspots: the three identified for May–June (which in fact represent four hotspots since the large concentration area near 37°N consists of two separate aggregation regions), plus one near 41°N (HS1–HS5 in Figure 2 and Table S2). The southernmost hotspot near 35°N (HS1) peaks the earliest (mid‐May) and has the lowest intensity, but also the largest interannual variability (standard deviation) and annual trend. During 1990–2010, the trend at HS1 accounts for a cumulative increase in krill concentrations of approximately half the peak intensity and twice the standard deviation. While HS1 intensity has steadily increased, its duration has shortened over the same period by roughly half a month, which is comparable to the timescale of interannual variations. However, the decrease in duration is outpaced by the increase in peak intensity, leading to an overall increasing trend, albeit slower, in annual mean krill concentrations at HS1.

The large area of high krill concentrations in the central region (36.5–38°N) represents two distinct hotspots: one south of 37°N (HS2) and one north of 37°N (HS3). Although the two hotspots have similar means, standard deviations, and trends in their intensity, the subregion south of 37°N experiences peak krill concentrations 3 weeks earlier than the subregion north of 37°N (Table S2). Interannual shifts in their duration and peak timing are also largely uncorrelated. The increasing trend in intensity at HS2 and HS3 is smaller relative to the mean than at HS1, yet the cumulative effect of the trend over the 21‐year period still exceeds interannual variability.

Two main aggregation regions occur in the northern part of the domain, the first peaking at 39.5–40°N during the first half of June (HS4) and the second reaching maximum intensity approximately a month later near 41°N (HS5). The earlier peak time of HS4 suggests that this hotspot may be sustained by northward advection of nearshore nutrients from the region of enhanced upwelling near 39°N (Fiechter et al., 2018). While both locations have similar mean intensities, interannual fluctuations in their properties are not strongly correlated and year‐to‐year intensity variation (standard deviation) near 41°N (HS5) is roughly twice that at 39.5–40°N (HS4). However, HS4 displays the largest year‐to‐year variations in the timing of peak intensity among all five hotspots (standard deviation of ~1 month). Although annual mean duration is rather uniform across all hotspots and ranges between 2 and 2.5 months, the northernmost hotspot (HS5) tends to be most persistent (Table S2).

### 3.3 Environmental Variability Associated With Krill Hotspots

The disparate range of responses (i.e., means, trends, and interannual variability) across hotspots suggests that dynamics controlling hotspot formation are strongly heterogeneous along the coast. Hence, biophysical processes determining the duration, intensity, and timing of krill aggregations must be understood locally in the context of regional patterns. To that effect, an EOF analysis is used to isolate dominant patterns of alongshore krill hotspot variability and to identify specific environmental conditions associated with these modes.

**Figure 3:**
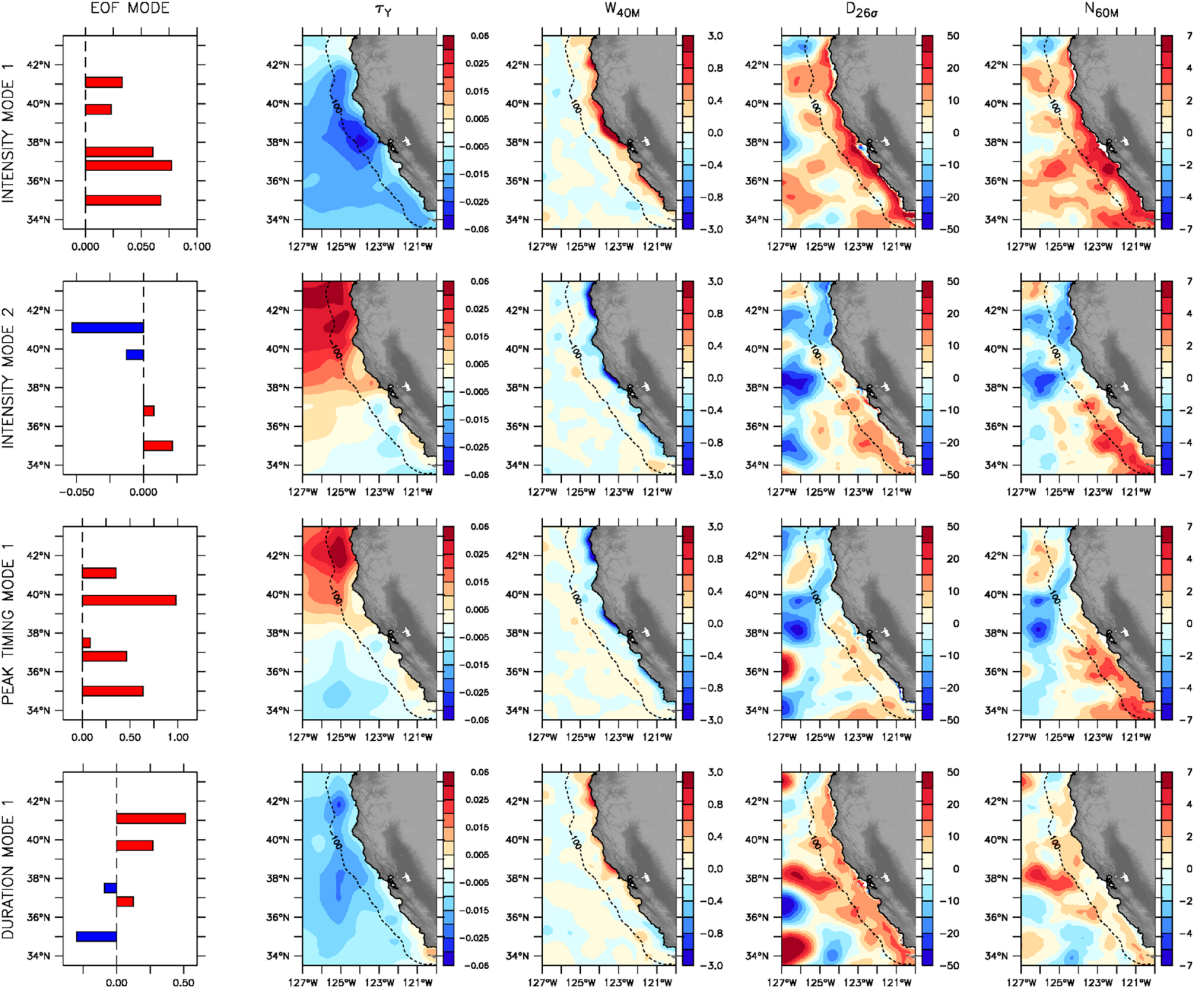
Environmental patterns associated with krill hotspot variability. Alongshore empirical orthogonal function (EOF) spatial mode (far left) and corresponding anomalies in (from left to right) meridional wind stress (N/m^2^), vertical velocity at 40 m (m/day), depth of 26.0 isopycnal (m), and nitrate at 60 m (mmolN/m^3^) for (from top to bottom) first and second EOF modes for intensity (67 and 15% of explained variance), first EOF mode for peak timing (21% of explained variance), and first EOF mode for duration (32% of explained variance). The dashed contour line indicates 100 km offshore.

The leading mode of variability for hotspot intensity (67% of explained variance) is a synchronous change (i.e., increase or decrease) in krill concentrations at all five alongshore locations (Figure 3, top row), with the magnitude of the response intensified in the southern part of the domain. Not unexpectedly, periods of increased hotspot intensity coincide with a region‐wide strengthening of upwelling favorable alongshore winds, shallower isopycnals, and enhanced delivery of nutrients near the coast. Based on the temporal amplitude of the first EOF mode, krill hotspots in the CCE were notably more intense in 1991 and 2008 and weaker in 1992, 1997–1998, 2005–2006, and 2009 (Figure S3).

The second mode of variability for hotspot intensity (15% of explained variance) is an asynchronous alongshore response, where the regions north and south of Point Arena (~39°N) vary out of phase with maximum amplitudes at the southern and northern most latitudes (i.e., near 35 and 41°N; Figure 3, second row). This pattern is predominantly associated with changes in surface wind stress in the northern part of the domain, resulting in alongshore differences in isopycnal depths and nutrient concentrations. Reduced upwelling in the north (i.e., weaker alongshore winds) leads to relatively deeper isopycnals near the coast north of ~39°N, with a corresponding alongshore nutrient gradient at 60‐m depth. The combination of weaker vertical velocities and lower nutrient concentrations below the mixed layer acts to limit production at the northern hotspot locations compared to the central and southern ones. Based on the temporal amplitude of the second EOF mode, krill hotspots in the southern CCE were notably more intense in 1993, 1997, and 2010 and weaker in 1998 and 2000–02 relative to krill hotspots in the northern CCE (Figure S4).

The dominant EOF mode for the timing of peak krill concentrations (21% of explained variance) exhibits low‐frequency variability correlated to the second EOF mode for hotspot intensity and, consequently, both modes are associated with similar patterns of environmental variability. However, while alongshore variations in intensity are out of phase, those for peak timing are synchronous throughout the region (Figure 3, third row). These results suggest that interannual variations in peak timing are related to those in hotspot intensity, and years for which peak krill concentrations occur later in the upwelling season coincide with an increase in hotspot intensity in the southern part of the domain and decrease in the northern part.

While the leading modes of variability for hotspot intensity and peak timing are both synchronous alongshore, that for duration (32% of explained variance) indicates an out‐of‐phase response between the southernmost hotspot near 35°N and the northern ones near 39.5–40°N and ~41°N (Figure 3, bottom row). Since anomalies in isopycnal depths and subsurface nutrient concentrations are synchronous coast‐wide, this pattern is predominantly associated with an alongshore gradient in upwelling favorable winds, enhancing coastal upwelling intensity in the northern part of the domain and reducing it between 34 and 36°N.

## 4 Discussion

**Figure 4:**
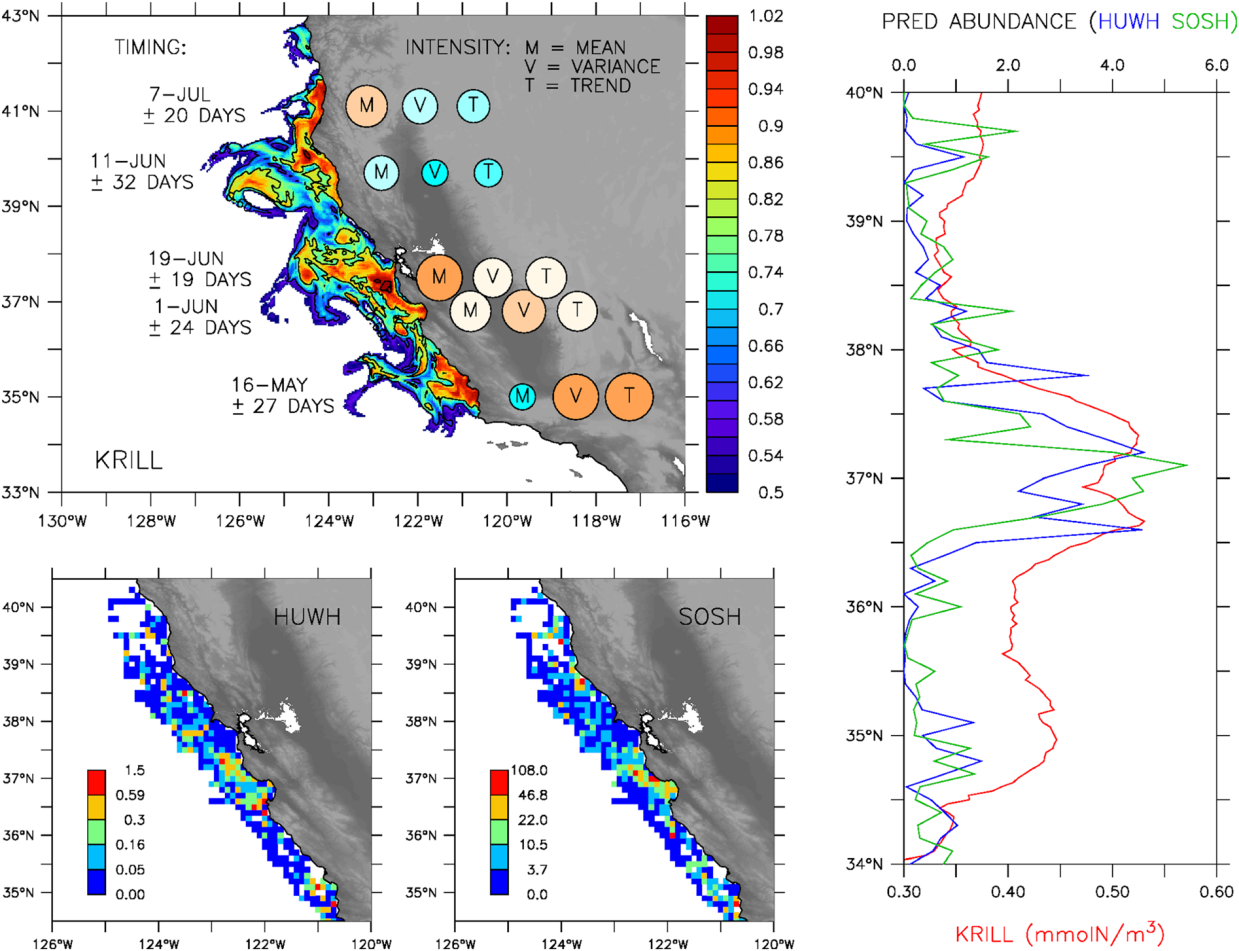
Ecosystem hotspots in the California Current Ecosystem. Top left: Simulated krill aggregations and variability in their timing and intensity during 1990–2010; circle size and color indicate relative mean (M), variance (V), and trend (T) in intensity across locations. Bottom: Observed distributions of humpback whale (HUWH) (left) and sooty shearwater (SOSH) (center) during May–June 1996–2018. Right: Zonally averaged (0–60 km offshore) krill concentrations (red; mmolN/m^3^) and predator abundances (blue = HUWH; green = SOSH) normalized by their meridional (34–40°N) mean.

Observing and predicting the formation and persistence of krill and higher trophic level species hotspots is difficult, and the results presented here offer insight into fundamental processes shaping lower trophic level ecosystem response in the CCE across space and time. Not only does the model recreate the location of known krill hotspots and their relationship to upwelling centers but it also explains their spatial variability and phenological properties in relation to alongshore variation in primary productivity and advection by near‐surface ocean currents (Figure 4, top left panel). The simulation further indicates that the magnitude of interannual variability and long‐term trend decreases with latitude, with the southernmost hotspot (HS1) experiencing the most extreme values. This finding is consistent with analyzed variability from satellite altimetry demonstrating that the southern CCE responds predominantly on interannual timescales and the northern CCE on intraseasonal timescales (Chavez & Messié, 2009).

The low‐frequency variability and long‐term trend in simulated krill concentrations are generally supported by ecosystem assessment surveys, which found that krill abundances were significantly lower throughout the 1990s (characterized by a positive PDO phase and strong El Niño events in 1992 and 1997–1998) and overall higher during the cooler decade of the 2000s (Ralston et al., 2015; Santora et al., 2014; Sydeman et al., 2013, 2015). Furthermore, the region‐wide decline in hotspot intensity predicted by the model during 2005–2007 corresponds to a previously identified period of delayed upwelling and intrusions of warm oceanic water, with known impacts to krill predators, such as reproductive failure of seabirds, distribution shifts of baleen whales and poor ocean salmon survival, and later recruitment (Fiechter et al., 2015; Fleming et al., 2016; Lindley et al., 2009; Sydeman et al., 2006).

Independent observations of the abundance and distribution of sooty shearwaters (*Ardenna grisea*) and humpback whales (*Megaptera novaeangliae*) collected during synoptic ecosystem surveys in May–June 1996–2018 closely resemble simulated patterns of krill hotspots within the central CCE (notably near 35°N and 36–38°N), which not only lends further confidence in the model's ability to reproduce krill aggregation locations but also suggests that these hotspots are indicative of broader ecosystem phenomena (Figure 4). The structural relationship between simulated krill aggregations and observed seabird and whale distributions implies that the model may be valuable for informing regional productivity assessments and ecosystem‐based management (PFMC, 2008; Lester et al., 2010; Maxwell et al., 2015), including linkages to populations of krill predators that are either resident breeders or migrate throughout the CCE in search of krill hotspots (Adams et al., 2012; Bailey et al., 2009; Calambokidis et al., 2015; Irvine et al., 2014; Redfern et al., 2013).

Finally, the modeling framework presented here can be extended to other krill‐centric marine ecosystems to facilitate a cross‐regional evaluation of processes governing krill hotspot formation and persistence. For instance, since local alongshore topographic impacts on upwelling intensity and productivity modulated by regional circulation patterns and basin‐scale influences are ubiquitous features of eastern boundary current upwelling regions, similar considerations on fundamental processes controlling krill aggregations and ecosystem hotspots should equally apply to the Benguela, Canary, and Humboldt Current Systems.

## Supporting information



Supporting Information S1
