# Supplementary material for: Krill Hotspot Formation and Phenology in the California Current Ecosystem
Source: Geophys Res Lett. 2020 Jun 28;47(13):e2020GL088039. doi: 10.1029/2020GL088039 (PMC7380319; doi:10.1029/2020GL088039)
Supplement: Supplementary file 1 — Supporting Information S1 [file GRL-47-e2020GL088039-s001.pdf]

AGU PUBLICATIONS

Geophysical Research Letters

Supporting Information for

Krill hotspot formation and phenology in the California Current Ecosystem

Jerome Fiechter[1], Jarrod A. Santora[2,3], Francisco Chavez[4], Devon Northcott[4,5], Monique Messié[4]

[1]Ocean Sciences Department, University of California at Santa Cruz, Santa Cruz, California, U.S.A.

[2]Fisheries Ecology Division, Southwest Fisheries Science Center, National Marine Fisheries Service, National Oceanic and Atmospheric Administration, Santa Cruz, California, U.S.A.

[3]Department of Applied Mathematics, University of California at Santa Cruz, Santa Cruz, California, U.S.A.

[4]Monterey Bay Aquarium Research Institute, Moss Landing, California, U.S.A

[5]now at Scripps Institution of Oceanography, University of California San Diego, California, U.S.A.

Contents of this file

Supplementary Table 1
Supplementary Table 2
Supplementary Figure 1
Supplementary Figure 2
Supplementary Figure 3
Supplementary Figure 4

Governing equation for krill (predatory zooplankton) in NEMUCSC
$$\underbrace{\frac{\partial Z_P}{\partial t}}_{\substack{time\ rate\\ of\ change}} + \underbrace{\vec{v}\cdot\nabla Z_P}_{\substack{advection\\ (from\ ROMS)}} \underbrace{- K_H\nabla_H^2 Z_P - K_V\frac{\partial^2 Z_P}{\partial z^2}}_{\substack{mixing\\ (from\ ROMS)}} = \underbrace{Q_{bio}}_{\substack{biological\\ sources\\ and\ sinks}}$$ $$Q_{bio} = \underbrace{\beta Z_P\left[\frac{G_{PL}P_L^2}{K_{PL}^2+P_L^2}+\frac{G_{ZL}Z_L^2}{K_{ZL}^2+Z_L^2}\right]}_{growth} - \underbrace{MZ_P^2}_{mortality}$$ Z_P: krill concentration (mmolN/m^3)<br>P_L: diatom concentration (mmolN/m^3)<br>Z_L: mesozooplankton concentration (mmolN/m^3)
**Parameterization of biological sources and sinks**
Krill grazing rate on diatom at 0°C: G_{PL} = 0.3 1/day<br>Half-saturation constant for diatom grazing: K_{PL} = 0.45 mmolN/m^3<br>Krill grazing rate on mesozooplankton at 0°C: G_{ZL} = 0.1 1/day<br>Half-saturation constant for mesozooplankton grazing: K_{ZL} = 0.9 mmolN/m^3<br>Krill growth efficiency: β = 0.3<br>Krill mortality rate at 0°C: M = 0.08 m^3/mmolN/day<br>(Temperature dependent rates have a Q_{10} of 2)

Supplementary Table 1. Governing equation and parameter selection for predatory zooplankton functional group in NEMUCSC.

**Krill Hotspot Property**	**Annual Mean and SD**	**Annual Trend**
Intensity	mmolN/m^3	mmolN/m^3/year
HS1	0.45 ± 0.105	0.011
HS2	0.53 ± 0.097	0.007
HS3	0.56 ± 0.083	0.008
HS4	0.50 ± 0.038	0.002
HS5	0.53 ± 0.069	0.004
Duration	months	months/year
HS1	2.32 ± 0.51	-0.024
HS2	2.32 ± 0.51	-0.012
HS3	2.42 ± 0.47	0.012
HS4	2.30 ± 0.53	0.019
HS5	2.50 ± 0.63	-0.008
Peak Timing	yearday	month/year
HS1	16 May ± 27 days	0.025
HS2	1 June ± 24 days	0.013
HS3	19 June ± 19 days	0.006
HS4	11 June ± 32 days	-0.019
HS5	7 July ± 20 days	0.018

Supplementary Table 2. Mean, standard deviation and annual trend of simulated krill hotspot properties. Hotspot locations are defined as 0.5° latitudinal bands centered at 35°N for HS1, 36.8°N for HS2, 37.5°N for HS3, 39.7°N for HS4 and 41.1°N for HS5 (see Fig. 2). Hotspots are identified as local alongshore peaks in krill abundance based on zonally averaged (0-100km offshore) values, limited yearly to periods when monthly mean concentrations exceed the annual mean plus one standard deviation.

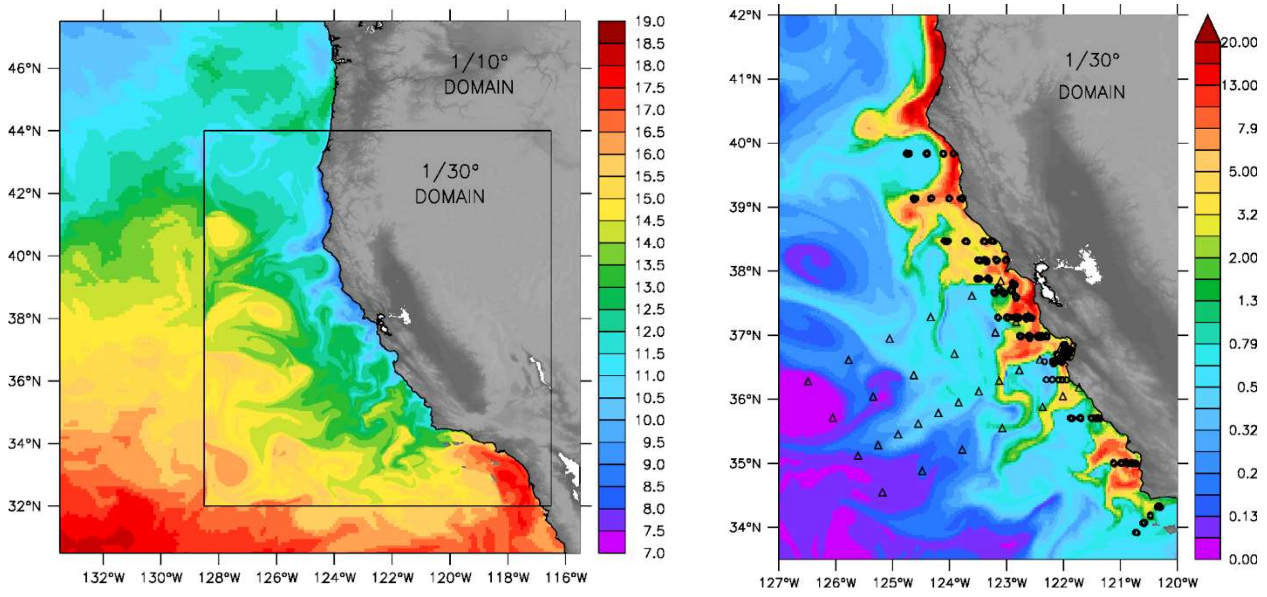

Supplementary Figure 1. ROMS-NEMUCSC model domain and locations of in situ krill observations. Left: dynamically downscaled sea surface temperature (°C) on 29 May 2007 from the 1/10° outer California Current domain and the 1/30° nested domain (black outline). Right: representative RREAS stations (black circles) superimposed on simulated surface chlorophyll (mg/m^3) on 29 May 2007 from the 1/30° nested domain (note that prior to 2004, the RREAS cruises only surveyed 36.5 to 38°N); triangles indicate locations of CalCOFI stations used by Lavaniegos and Ohman (2007) to calculate euphausiid abundance for the central CCE.

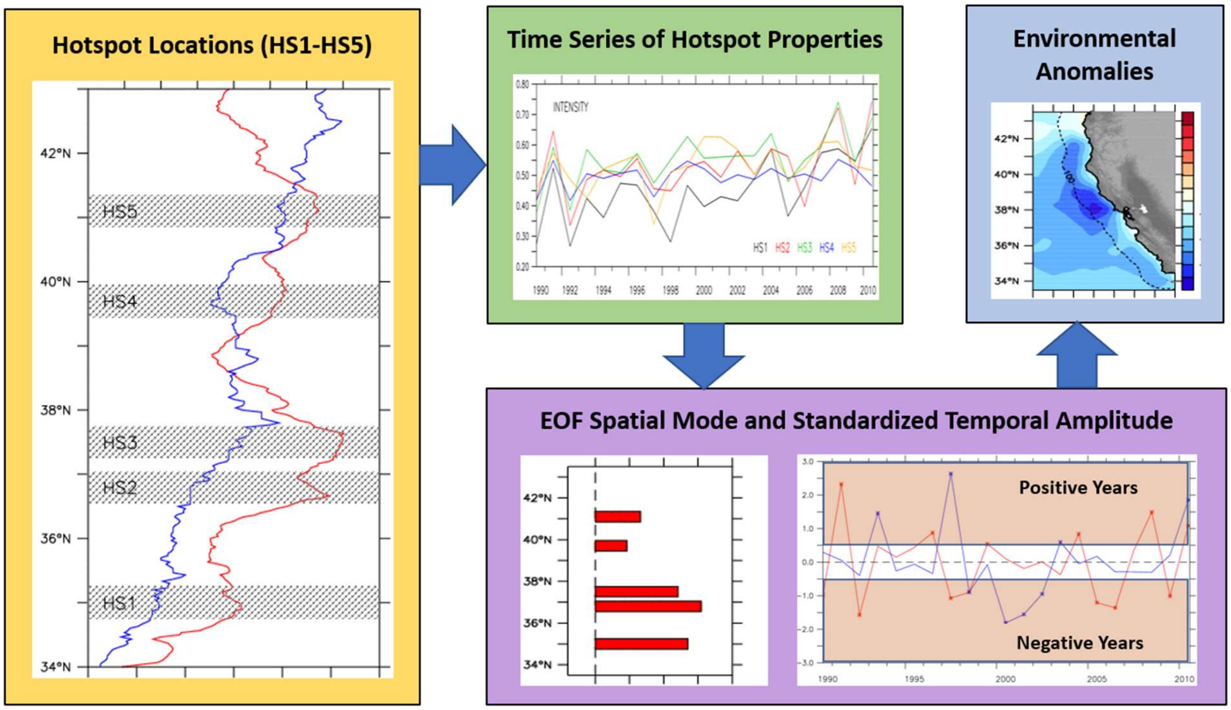

Supplementary Figure 2. EOF analysis for krill hotspot variability and corresponding environmental anomalies. Step 1 (yellow box): hotspots are identified as 0.5° alongshore band corresponding to local peak in krill concentrations. Step 2 (green box): annual mean hotspot properties (intensity, duration and timing) are calculated at each hotspot location. Step 3 (purple box): for each property, time series at all hotspot locations are combined into an EOF analysis to extract the dominant modes of alongshore variability and their standardized temporal amplitudes. Step 4 (blue box): environmental anomalies for selected variables (alongshore winds, vertical velocities, isopycnal depth, and nitrate concentrations) are calculated by subtracting the mean over all negative amplitude years (< 0.5 standard deviation) from the mean over all positive amplitude years (> 0.5 standard deviation).

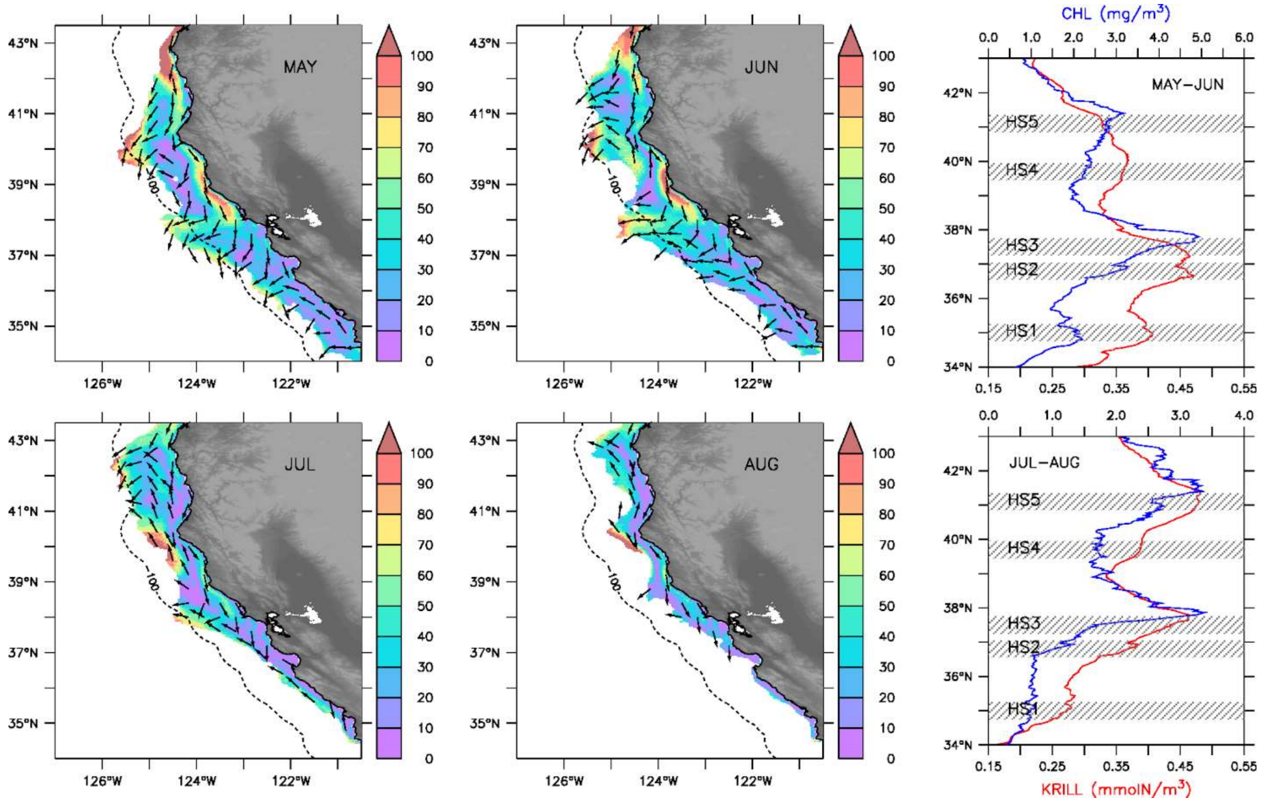

Supplementary Figure 3. Seasonal advection and krill hotspot locations. Left and center panels: simulated horizontal advection distance (km; colorscale) and direction (vectors) by near surface currents (0-40 m depth) over a krill doubling period (~1 week) during May, June, July and August (dashed contour line indicates 100 km offshore). Right: simulated mean nearshore surface chlorophyll (blue; mg/m^3) and krill (red; $mmolN/m^3$) concentrations during May-June (top) and July-August (bottom); shaded 0.5° bands denote alongshore extent of five seasonal peaks identified as local hotspots in simulated surface krill concentrations averaged 0-100 km offshore.

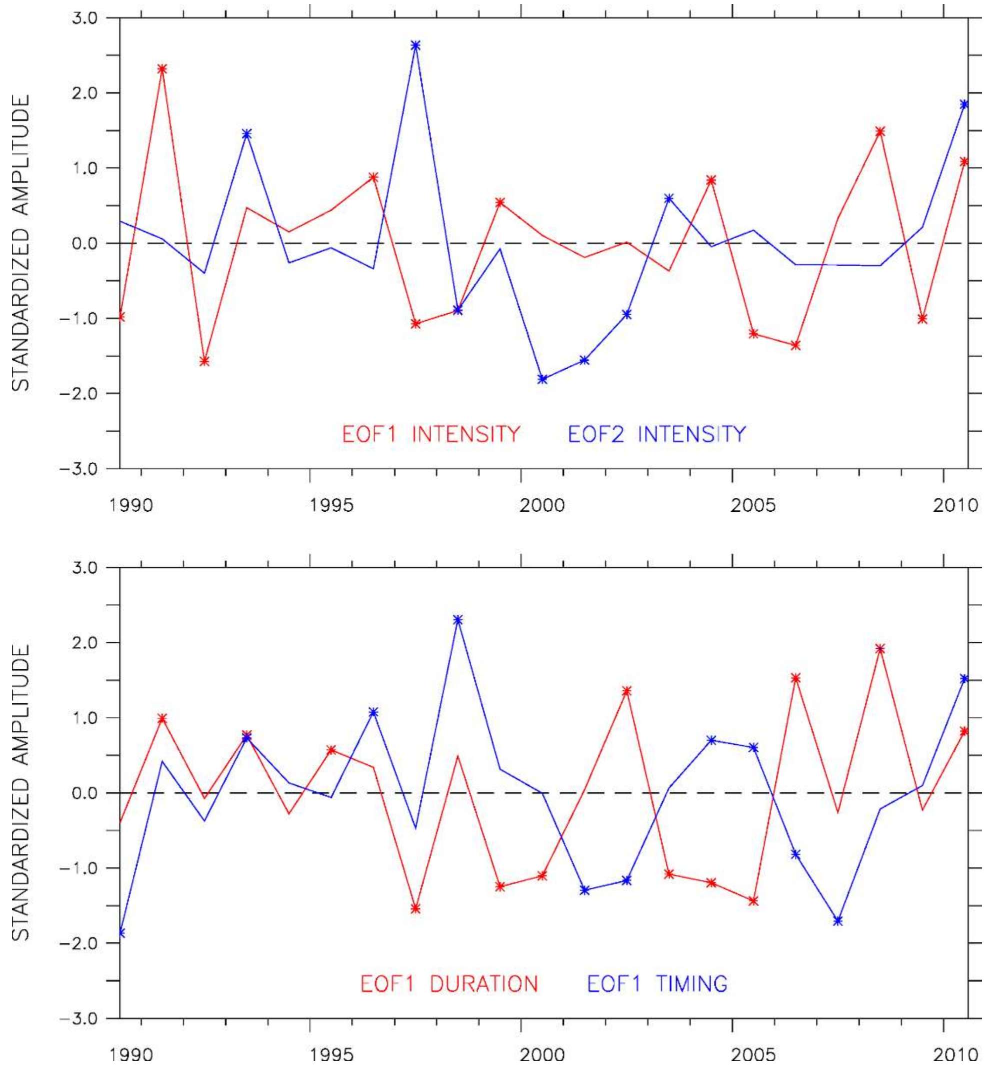

Supplementary Figure 4. Standardized temporal amplitude of EOF modes. Left: amplitudes of the first (red) and second (blue) EOF modes for krill hotspot intensity. Right: amplitudes of the first EOF modes for krill hotspot duration (red) and peak timing (blue). Symbols indicate years for which the amplitude is above or below 0.5 standard deviation.
